# Influence of Obesity and Metabolic Disease on Carotid Atherosclerosis in Patients with Coronary Artery Disease (CordioPrev Study)

**DOI:** 10.1371/journal.pone.0153096

**Published:** 2016-04-11

**Authors:** Eva Talavera-Garcia, Javier Delgado-Lista, Antonio Garcia-Rios, Nieves Delgado-Casado, Purificacion Gomez-Luna, Angela Gomez-Garduño, Francisco Gomez-Delgado, Juan F. Alcala-Diaz, Elena Yubero-Serrano, Carmen Marin, Ana I. Perez-Caballero, Francisco J. Fuentes-Jimenez, Antonio Camargo, Fernando Rodriguez-Cantalejo, Francisco J. Tinahones, Jose M. Ordovas, Francisco Perez- Jimenez, Pablo Perez-Martinez, Jose Lopez-Miranda

**Affiliations:** 1 Lipid and Atherosclerosis Unit. IMIBIC/Reina Sofia University Hospital/University of Cordoba, and CIBER Fisiopatologia Obesidad y Nutricion (CIBEROBN), Instituto de Salud Carlos III, Madrid, Spain; 2 Biochemical Laboratory, Hospital Universitario Reina Sofia, Cordoba, Spain; 3 Biomedical Research Laboratory, Endocrinology Department, Hospital Virgen de la Victoria, Malaga, and CIBER Fisiopatologia Obesidad y Nutricion (CIBEROBN), Instituto de Salud Carlos III, Madrid, Spain; 4 Nutrition and Genomics Laboratory, JM-USDA Human Nutrition Research Center on Aging at Tufts University, Boston, MA, United states of America; 5 Department of Cardiovascular Epidemiology and Population Genetics, Centro Nacional de Investigaciones Cardiovasculares (CNIC), Madrid, IMDEA, Spain; Medical University Hamburg, University Heart Center, GERMANY

## Abstract

**Background:**

Recent data suggest that the presence of associated metabolic abnormalities may be important modifiers of the association of obesity with a poorer prognosis in coronary heart disease. We determined the influence of isolated overweight and obesity on carotid intima media thickness (IMT-CC), and also assessed whether this influence was determined by the presence of metabolic abnormalities.

**Methods:**

1002 participants from the CordioPrev study were studied at entry. We determined their metabolic phenotypes and performed carotid ultrasound assessment. We evaluated the influence of obesity, overweight and metabolic phenotypes on the IMT-CC.

**Results:**

Metabolically sick participants (defined by the presence of two or more metabolic abnormalities) showed a greater IMT-CC than metabolically healthy individuals (p = 4 * 10^−6^). Overweight and normal weight patients who were metabolically healthy showed a lower IMT-CC than the metabolically abnormal groups (all p<0.05). When we evaluated only body weight (without considering metabolic phenotypes), overweight or obese patients did not differ significantly from normal-weight patients in their IMT-CC (p = 0.077). However, obesity was a determinant of IMT-CC when compared to the composite group of normal weight and overweight patients (all not obese).

**Conclusions:**

In coronary patients, a metabolically abnormal phenotype is associated with a greater IMT-CC, and may be linked to a higher risk of suffering new cardiovascular events. The protection conferred in the IMT-CC by the absence of metabolic abnormality may be blunted by the presence of obesity.

**Trial Registration:**

ClinicalTrials.gov NCT00924937

## Introduction

Cardiovascular disease (CVD) is one of the leading causes of morbidity and mortality in Western countries. Despite the fact that advances in the knowledge of its pathophysiological and etiological factors have decreased patient mortality with the use of clinical practice guidelines, up to 30% of patients with coronary artery disease have new events[1–3]. In the search for new pathogenic factors of the disease, it has been suggested that the coexistence of obesity in these patients may worsen the prognosis of coronary artery disease[4–6]. However, these latter results have not been uniform in all studies, since it seems that only certain populations of obese patients have an increased cardiovascular risk, and that not all obese individuals exhibit uniformity in terms of metabolic and cardiovascular risk[7–9]. Furthermore, it has been observed that populations with similar BMI may have a very different behavior in terms of their factors of metabolic and cardiovascular risk[8–11]. To sum up, current knowledge raises the possibility that it is not obesity itself, but certain clinical phenotypes often associated with it, which could explain the associated increased cardiovascular risk attributed to obese persons.

One of the current lines of research in this field is to determine the factors that differentiate each of the metabolic phenotypes[8, 9, 12–16]. Six types of metabolic phenotypes according to their body mass index and the presence or absence of metabolic disease have been described in the general population[17]. Among them, the so-called *metabolically healthy obese* phenotype (MHO) is worthy of note, characterized by having increased fat mass, but high levels of HDL-c and good insulin sensitivity, accompanied with normal serum levels of glucose and triglycerides and therefore a more favorable cardiovascular profile[14]. When comparing this phenotype to others, this group appears to have an intermediate cardiovascular risk between healthy, normal-weight subjects and obese patients with metabolic disease[11, 14, 18].

Contrasting with these *healthy obese* individuals, the so-called *metabolically sick normal weight* (MSNW) is a group that includes individuals of normal weight, but with early signs of insulin resistance, hyperinsulinemia, atherogenic dyslipidemia and hypertension, with a high susceptibility to developing type 2 diabetes mellitus and cardiovascular disease [7–9, 19]. The individuals with this phenotype may be undiagnosed, because they have only mild cardiovascular symptoms, and the “healthy” appearance derived from their normal body mass index means that they do not go to their physician for a general check-up. Identification and treatment of the metabolic disorders in these persons, however, could be effective in the primary prevention of obesity and/or associated cardiovascular diseases[20].

In summary, and although, theoretically, it has been proposed that different metabolic phenotypes may influence the development/prognosis of coronary artery disease, there are currently few studies investigating whether these phenotypes are associated with differences in markers of the extent of the disease.

Our objective was to determine the influence of isolated overweight and obesity on the carotid intima media thickness (IMT-CC), a marker that has been validated as a predictor of the prognosis of coronary artery disease[3, 21, 22], and also to assess whether this influence was affected by the presence of metabolic disease.

## Materials and Methods

### Population

This work has been carried out in the context of the CORDIOPREV study. The CORDIOPREV study is a prospective, randomized, controlled trial that includes 1,002 patients with coronary heart disease, who had their last coronary event over six months before joining the study. Participants have been divided into two different models of diet (Mediterranean and low-fat) which they follow for a period of seven years, along with conventional treatment for coronary heart disease. Patients were recruited from November 2009 to February 2012, mostly in the Reina Sofia University Hospital (Cordoba, Spain), but patients from other hospitals in the province of Cordoba and Jaen were also admitted. The study was approved by the Ethics Committee for Clinical Investigations of the Reina Sofía University Hospital in Cordoba, and participants gave their informed consent in writing to join the study. The inclusion and exclusion criteria have been published elsewhere, and are also reported in Table 1 [23]. In short, patients were eligible if they were between 20 and 75 years old, with established coronary artery disease without clinical events in the last six months, with the intention of participating in a long-term monitoring study, with no other serious illnesses and if they had a life expectancy of at least five years.

**Table 1:** Inclusion and exclusion criteria for CORDIOPREV study.

**Ages Eligible**	20 to 75 Years
**Genders Eligible**	Both
**Inclusion Criteria**	Unstable coronary disease
	Acute Myocardial Infarction
	Unstable Angina
	Chronic Coronary Disease at high risk for event
**Exclusion Criteria**	Age <20 or >75 years (or life expectancy lower than 5 years)
	Patients already planned for revascularization
	Patients submitted to revascularization in the last 6 months
	Grade II/IV Heart failure
	Left ventricle dysfunction with ejection fraction lower than 35%
	Patients unable to follow a protocol
	Patients with severe uncontrolled Diabetes Mellitus, or those with Renal Insufficiency with permanent plasma creatinine higher than 2 mg/dl, or cerebral complications of Diabetes Mellitus
	Other uncontrolled chronic diseases, including: Psychiatric diseases, Chronic Renal Insufficiency, Chronic Hepatopathy, Active Malignancy, COPD, Diseases of the digestive tract or Endocrine disorders
	Patients participating in other clinical trials (at the time of enrollment or 30 days prior to enrollment)

For the specific aims of this work, the patients were classified according to the presence or absence of the six cardiometabolic abnormalities described below, based on Wildman et al[17]. From the initial sample of 1,002 subjects, in this article we included only the 939 subjects with all the carotid ultrasound data, analytical and anthropometric data. The causes of the absence of data for the remaining 63 patients were as follows: 37 refused to conduct the echography, 14 withdrew from the study before conducting the tests, 12 other causes.

### Cardiometabolic criteria

The cardiometabolic abnormalities used were those proposed by Wildman[17]. For the definition of the homeostasis model of insulin resistance (HOMA-IR), we used the one adapted to the Spanish population[24] and for the range of C-reactive protein (CRP) defining a high risk, we used the guidelines suggested by CDC / AHA[25].

Thus, the cardiometabolic criteria initially proposed were as follows:

- Elevation of systolic / diastolic blood pressure ≥ 130/85 mmHg or use of anti-hypertensive therapy
- High fasting triglyceride levels ≥ 150 mg / dl
- Low HDL- C: HDL -C <40 mg / dl in men or < 50 mg / dl in women or use of lipid-lowering therapy
- High levels of fasting glucose: ≥ 100 mg / dl or use of anti-diabetic treatment
- Insulin resistance: HOMA -IR > 2.6
- Presence of systemic inflammation: CRP ≥ 3mg/dl.

In our study, we made a change in the assessment of two factors (low HDL or taking anti-hypertensive or lipid-lowering drugs), since almost all coronary patients receive these two treatments, according to the current treatment guidelines. Instead, we identified patients in the lower tertile of HDL and in the upper tertile of systolic blood pressure, and we could infer in our sample those with the worst figures in these two variables.

### Metabolic phenotypes

- Metabolically healthy normal weight: BMI < 25 and < 2 metabolic criteria
- Metabolically sick normal weight: BMI < 25 and ≥ 2 metabolic criteria
- Metabolically healthy overweight: BMI 25–30 and < 2 metabolic criteria
- Metabolically sick overweight: BMI 25–30 and ≥ 2 metabolic criteria
- Metabolically healthy obese: BMI > 30 and <2 metabolic criteria
- Metabolically sick obese: BMI > 30 and ≥ 2 metabolic criteria

### Intima media thickness assessment

Intima media thickness was assessed at the start of the study. All patients were examined in supine position with the neck hyperextended and with the chin turned to the side. The carotid arteries were examined bilaterally using a Doppler ultrasound high-resolution B-mode (Envisor C Ultrasound System, Phillips, USA), following the recommendations of the American Society of Echocardiography Carotid Intima-Media Thickness Task Force [26]. The observers were unaware of the participants’ demographic and cardiovascular risk data during the assessment. The measurements were taken using semi-automatic software (QLAB Advance Ultrasound Quantification Software, v5.0, Phillips, USA)). Three measurements were taken for each patient, and we obtained the general mean of the intima-media thickness of both common carotid arteries (IMT-CC).

### Statistical analysis

The variables used in this article were assessed for normality of distribution. Triglyceride values were normalized by log10 transformation in the analysis. Statistical analyses were carried out using SPSS 19.0 for Windows (SPSS Inc., Chicago, IL, USA). Three models of ANCOVA-based tests (with Bonferroni correction) were used to test for associations of IMT-CC with the presence of metabolic disease (yes/no), the presence of obesity (yes/no) and the different categories of BMI (<25; 25–30; >30). In the three ANCOVA tests, age, smoking habit and sex were included as covariates.

A second approach to the IMT-CC association with phenotypic variables was performed by combining metabolic phenotypes and weight, thus dividing the sample into 6 groups (metabolically healthy normal-weight, metabolically healthy overweight, metabolically healthy obese, metabolically sick normal-weight, metabolically sick overweight, metabolically sick obese). This ANCOVA test used the same modeling as above, using the 6 groups as the fixed factor, the IMT-CC as the dependent variable and age, smoking habit and sex as covariates. The effect size for comparison of obesity/no obesity and metabolic phenotypes was tested using both non-standardized (differences of means) and standardized (partial eta squared) methods.

Bivariate correlation (Spearman test) was used to test for the association of number of metabolic abnormalities and IMT-CC.

## Results

Table 2 shows the baseline characteristics of the population. As expected, the group of metabolically sick participants had higher systolic and diastolic blood pressure, triglycerides, fasting blood glucose, HOMA-IR and PCR as well as lower HDL-c. Notably, we did not find any differences in LDL-C.

**Table 2:** Baseline.

Variables (Units)	Metabolically Healthy (n 294)	Metabolically Sick (n 645)	p
Age (Years)	58.7±9.6	60±8.7	0.04
Men,%	84.4	84.2	0.935
BMI (kg/m^2^)	29.5±4.1	31.8±4.5	<0.001
Systolic Blood Pressure (mm Hg)	129.9±15.2	142.5±20.1	<0.001
Diastolic Blood Pressure (mm Hg)	74.4±9.8	78.4±11.2	<0.001
Heart rate (beats/min)	62.6±10.2	67.7±11.2	<0.001
Fasting Triglycerides (mg/dL)	97.2±42.1	148.7±91.9	<0.001
hs-CRP (mg/L)	1.6±1.3	2.8±1.8	<0.001
LDL-C (mg/dL)	85.4±22.9	83.6±25.0	0.317
Fasting Glucose (mg/dL)	92.3±13.4	123.8±45.0	<0.001
HDL-C (mg/dL)	46.7±9.8	39.9±9.1	0.091
Total cholesterol (mg/dL)	147.7±28.1	151.6±33.0	<0.001

Data are given as mean±SD unless otherwise specified. Abbreviations: BMI, body mass index; HDL-C, high-density lipoprotein cholesterol; LDL-C, low-density lipoprotein cholesterol; hs-CRP, high sensitivity C-reactive protein. Metabolically sick patients were diagnosed when they had two or more of: 1. High blood pressure: Highest tertile for systolic blood pressure; 2. High triglyceride level: Fasting triglyceride level ≥150 mg/dL; 3. Decreased HDL-C level: Lowest tertile for HDL-C; 4. High glucose level: Fasting glucose level ≥100 mg/dL or antidiabetic medication use; 5. Insulin resistance: HOMA-IR >3; 6. Systemic inflammation: hsCRP level >3mg/L.

**Fig 1:**
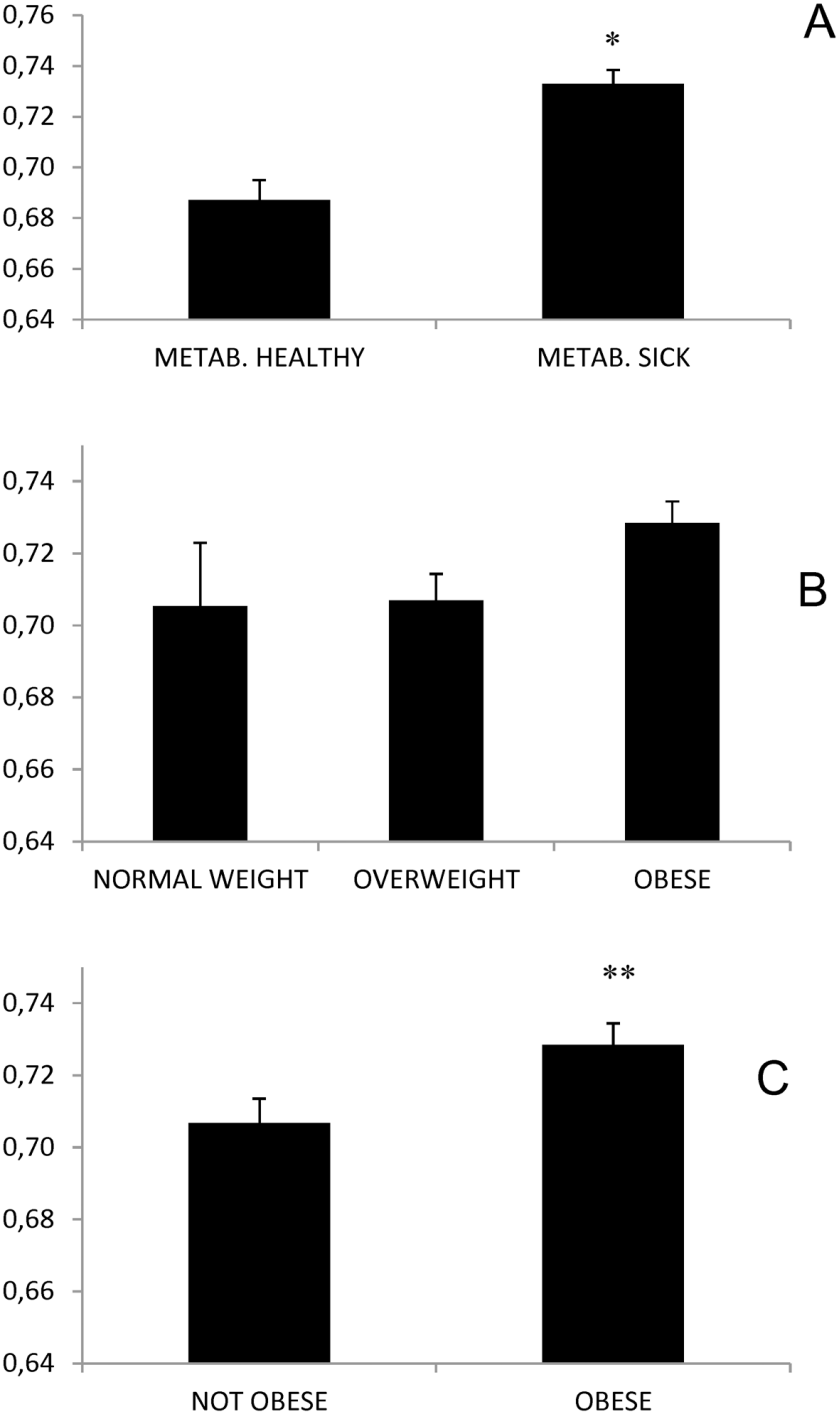
Influence of weight and metabolic disease on IMT-CC of coronary patients in the CordioPrev study. All data are Mean +/- SEM (mm). Panel A: IMT-CC of patients with and without metabolic disease (adjusted by age and gender). *p 1.7+10^−6^. Panel B: Influence of weight in three categories (normal, overweight and obese). Panel C: Influence of weight in two categories (not obese versus obese).** p 0.016.

Regarding the main objective of this study, IMT-CC was higher in metabolically sick participants (two or more than 2 defining abnormalities of metabolic phenotype) compared to the metabolically healthy (0.73 SEM 0.01mm vs 0.69 SEM 0.01mm, p = 4 * 10^−6^, Fig 1A). The number of abnormalities correlated with IMT-CC (p = 2*10^−7^ r 0.42, S1 Fig).

In our study, isolated overweight or obesity by itself was not a determinant of IMT-CC versus normal weight (normal weight 0.71 SEM 0.02mm, overweight 0.71 SEM 0.07mm, obese 0.73 SEM 0.06mm, p = 0.077, Fig 1B). When we grouped normal weight and overweight in a composite (not including obese), these participants had lower IMT-CC versus obese persons (0.71SEM0.07 versus 0.73 SEM 0.06mm, p = 0.016, Fig 1C). The effect sizes found were: mean difference 0.02mm; partial eta squared: 0.006.

**Fig 2:**
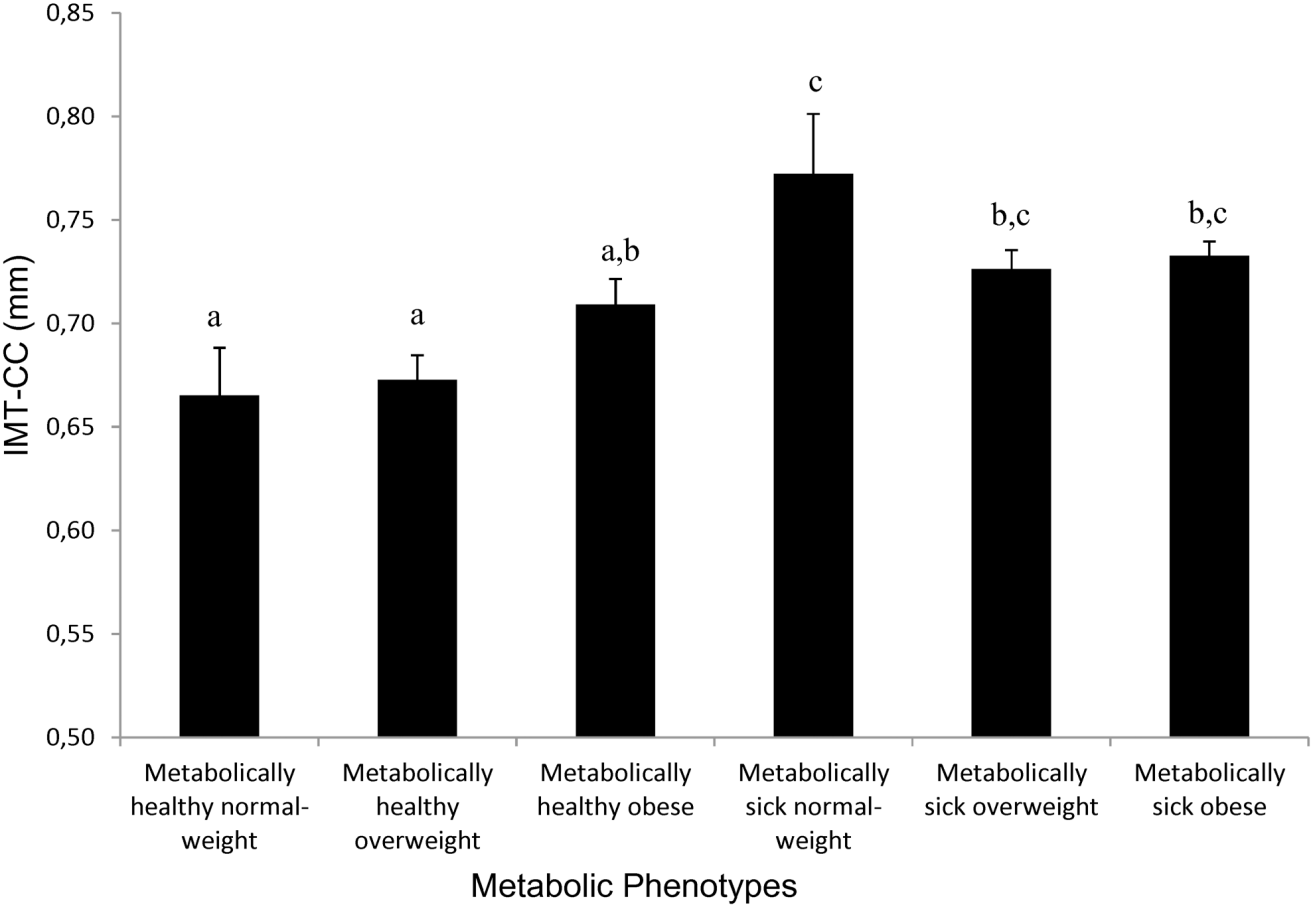
Influence of metabolic phenotypes on IMT-CC of coronary patients in the CordioPrev study. All data are Mean +/- SEM (mm). Columns which do not share at least one letter are different at p<0.05. Sample size for the different groups is as follows: Metabolically healthy normal-weight (n = 36); metabolically sick normal-weight (n = 22); metabolically healthy overweight (n = 136); metabolically sick overweight (n = 221); metabolically healthy obese subjects (n = 122); metabolically sick obese subjects (n = 402). Specific p values for the different significant comparisons are as follows: Metabolically healthy normal-weight subjects are different to metabolically sick normal-weight (p = 0.0036), metabolically sick overweight (p = 0.0134) and metabolically sick obese subjects (p = 0.0048); Metabolically healthy overweight subjects are different to metabolically sick normal-weight (p = 0.0014), metabolically sick overweight (p = 0.0004) and metabolically sick obese subjects (p = 0.0001).

When we combined phenotypes using weight and metabolic criteria, we observed that metabolically healthy, normal weight patients had a lower IMT-CC than all other groups (all p<0.02, Fig 2). The effect sizes found were: mean differences ranged from 0.053mm to 0.107mm; partial eta squared: 0.031.

However, the presence of isolated overweight or obesity (without metabolic disease) did not condition a greater IMT-CC compared with metabolically healthy normal weight. In fact, metabolically healthy, overweight patients kept the same difference with all groups of metabolically sick patients, including those who were normal weight. The metabolically healthy, obese showed an intermediate behavior, as they did not differ from any other groups. However, they did show a trend towards lower IMT-CC than metabolically sick patients (p = 0.043, p = 0.266 and p = 0.096 versus normal weight, overweight and metabolically sick patients respectively) (Fig 2).

We also analyzed the sample only looking at the presence or absence of obesity (BMI > 30) and presence or absence of metabolic disease, resulting in four groups (obese and not-obese subjects with and without metabolic disease). Not-obese, metabolically healthy had lower IMT-CC than not-obese metabolically sick (0.67 SEM 0.01 vs 0.73 SEM 0.01, P < 0.001). Not-obese, metabolically healthy patients also had lower IMT-CC than the obese, metabolically sick (0.67 SEM 0.01 vs 0.73 SEM 0.01, P < 0.001). As had happened when establishing the 6 phenotypes, healthy obese subjects (IMT-CC 0.71 SEM 0.01) showed an intermediate phenotype with a non-significant trend towards lower IMT-CC than metabolically sick participants (p = 0.157 and p = 0.096 versus not-obese and obese metabolically sick patients, respectively).

## Discussion

Our paper shows two main findings. First, that the existence of metabolic disease (defined by the existence of two or more metabolic abnormalities) is a clear determinant of IMT-CC in coronary patients. Second, that the existence of overweight and obesity aggravate IMT-CC in the presence of metabolic disease, but not when the increased body weight is not associated with metabolic disease.

The search for predictors of the evolution of coronary heart disease is an active field in cardiovascular research, because up to a third of coronary patients have clinical relapses, despite the advances in diagnosis and treatment of disease[1–3, 20]. Perhaps the therapeutic efforts made for people with coronary artery disease should be graded according to their real, attributable risk. In this scenario, it is increasingly important to identify factors that can help in the prognostic assessment of the disease beyond the classic criteria (hypertension, LDL, smoking, age and sex).

The IMT-CC is a measurement whose usefulness as a prognostic marker has been proved [3, 21, 22]. The recent interest in identifying and categorizing patients according to their cardiovascular risk has led us to use the measurement of IMT-CC as a marker of atherosclerosis that correlates with extensive coronary disease in adults and predicts future events[27]. Doppler ultrasound of the carotid arteries is a non-invasive tool and is considered effective for exploring and quantifying anatomical changes of IMT-CC, in which the intima, media, or both, suffer from increased hypertrophy to adapt to changes of blood flow, blood pressure and lumen diameter wall [27, 28]. Therefore, IMT-CC can be considered a marker of atherosclerosis that correlates with cardiovascular risk factors and the incidence of vascular diseases, such as myocardial infarction or stroke.

In our case, the existence of two or more markers of metabolic disease was associated with higher IMT-CC, but the importance of body weight was minor. Some recent studies have sparked a controversy over the role of obesity as a prognostic factor in patients with coronary artery disease[4–9], but, to our knowledge, this is the first large cohort study in this population to show that metabolic disease is much more closely associated with IMT-CC than weight itself. Although a consensus position of the AHA established the existence of obesity as a prognostic factor[29], data about the importance of overweight in this and other statements is not currently clear[30]. Furthermore, other studies have suggested that certain metabolic phenotypes often associated with obesity may be behind the additional, increased risk found [31, 32]. In this way, the so-called metabolic phenotypes have been established, combining the existence of metabolic abnormalities in the presence of overweight or obesity, in the general population[17]. In our study, we made an adjustment in the evaluation of two factors (low HDL or taking anti-hypertensive or lipid-lowering drugs), since almost all coronary patients receive these two treatments according to the protocol, and assessed these two markers with tertiles for HDL and systolic arterial pressure. Our results showed that the presence of metabolic disease *per se* is associated with a worse IMT-CC (with a p value of 10^−6^), and when we used metabolic phenotypes (the combined assessment of metabolic features and weight), we were able to identify a gradation of effects. Metabolically-healthy individuals with normal weight have a lower IMT-CC than all the other groups, and metabolically healthy, overweight people have a lower IMT-CC than any of the metabolically sick groups of patients (whatever their BMI). Healthy, obese patients seem to have an intermediate phenotype, in the sense that there are no differences in IMT-CC when compared to any metabolically-sick groups, although they do have a tendency to have a lower IMT-CC than the latter.

Our study also informs us about the behavior of overweight compared with obese subjects regarding IMT-CC. In all our comparisons, the overweight were not related to a worse IMT-CC compared to normal weight individuals. The comparison of the behavior of overweight versus obese persons is important, as general recommendations and documents related to the therapeutic approach of weight excess do not explain clearly whether any differences exist between these two phenotypes. Identifying similarities and differences between these two groups may help us to manage these two conditions in a satisfactory way [30].

Two main features (weight and metabolic abnormalities) have been tested in our study. Both features, obesity and metabolic disease, have been related to a higher IMT-CC, and, as discussed before, both interact to produce a worse IMT-CC. If we have to weigh up the particular contribution of each of these two factors, we consider that they are equal neither in significance nor extent. First, when evaluating the degree of excess weight in IMT-CC, it was only when we grouped normal-weight persons with overweight into a composite group of “non-obese subjects” that we found significant differences versus obese persons. However, when we performed an ANCOVA using the three defined categories (normal weight, overweight and obese), we did not find these differences. Second, the possibility of committing an alpha error when stating that metabolic disease is associated with a higher IMT-CC is much lower (p = 4 * 10^−6^) than when stating that it is obesity that is linked to it (p = 0.016). We also performed an estimation of the effect size in our comparisons, using non-standardized (differences of means) and standardized (partial eta squared) methods. The differences in not-obese versus obese were 0.02mm, while the differences in each pair of significant different groups in the metabolic phenotypes ranged from 0.053mm to 0.107mm (3 to 5 times higher differences between groups than in the obese/not obese comparisons). Partial Eta squared for the first comparison (obese/not obese) was 0.006, while it was 0.031 (five times higher) for the metabolic phenotypes. Based on these premises, we conclude that the contribution of metabolic abnormalities, at least in our study, is higher than that of body weight.

One intriguing result stands out in our study. The presence of metabolic disease in patients with normal weight seems to be associated with a tendency to have a higher IMT-CC (although not statistically significant) than patients with metabolic disease and overweight or obesity. In our opinion, there are two factors that could be involved here. On the one hand, the low frequency of this metabolic phenotype (there were only 22 normal-weight participants with metabolic disease against 221 overweight and 402 obese), and on the other, a possible process of natural selection. It is possible that being of normal weight means that there needs to be a greater degree of atherosclerosis in order to produce a coronary event. In other words, in these people, their arteries need to have a higher level of vascular disease to cause the existence of clinical coronary disease. Comparisons with similar studies are difficult, as there are few studies of these characteristics, and even fewer if we only include coronary patients. A 2014 systematic review aimed to explore cardiovascular risk in metabolically healthy obese subjects versus metabolically healthy normoweight individuals. Although the criteria for inclusion and categorizing of the patients was not equivalent to our study, they reached a conclusion that may support our findings. Metabolically healthy obese subjects were at some risk when compared to metabolically healthy normoweight individuals. Unfortunately, the other metabolic phenotypes were not explored[33]. Although it did not evaluate metabolic phenotypes, a recent work in 100 coronary patients showed that some of the factors used for defining the metabolic phenotypes (such as hypertension, LDL or C-reactive protein) were associated with higher carotid atherosclerotic parameters[28].

The criteria for defining metabolic abnormalities include markers of altered glucose and lipid metabolism, blood pressure and inflammation. In fact, the link between IMT-CC and inflammation is so well established that it has even been proposed as a marker of the status of inflammatory diseases[34–39]. The recent European Society of Cardiology Guidelines on diabetes, pre-diabetes and cardiovascular diseases revises and summarizes how all of the criteria for defining metabolic disease have been separately linked to higher inflammation, oxidative stress or vascular inflammation. It is logical to think that the combination of all of them together could promote the increase of IMT-CC by these pathways [3], a conjecture which is consistent with our findings.

Our study was carried out at the enrollment of the patients in the CordioPrev study, a prospective study to evaluate the clinical response of about 1000 patients when given a long-term dietary intervention with two healthy dietary patterns (low-fat and Mediterranean). Although the CordioPrev study may be able to evaluate in the near future the influence of metabolic phenotypes in the onset of cardiovascular events and clinical outcomes, this article serves to demonstrate that, at least, metabolic disease appears to be associated with higher IMT-CC, and therefore to a worse vascular condition. In our case, obesity seems to be an aggravating factor in the presence of metabolic disease.

## Supporting Information

S1 Fig IMT-CC of patients in the CordioPrev study depending on the number of metabolic abnormalities. (DOCX)
