# Correction: Influence of Obesity and Metabolic Disease on Carotid Atherosclerosis in Patients with Coronary Artery Disease (CordioPrev Study)

**DOI:** 10.1371/journal.pone.0157213

**Published:** 2016-06-09

**Authors:** Eva Talavera-Garcia, Javier Delgado-Lista, Antonio Garcia-Rios, Nieves Delgado-Casado, Purificacion Gomez-Luna, Angela Gomez-Garduño, Francisco Gomez-Delgado, Juan F. Alcala-Diaz, Elena Yubero-Serrano, Carmen Marin, Ana I. Perez-Caballero, Francisco J. Fuentes-Jimenez, Antonio Camargo, Fernando Rodriguez-Cantalejo, Francisco J. Tinahones, Jose M. Ordovas, Francisco Perez- Jimenez, Pablo Perez-Martinez, Jose Lopez-Miranda

There is an error in affiliation #1. The correct affiliation #1 should be: Lipid and Atherosclerosis Unit. IMIBIC/Reina Sofia University Hospital/University of Cordoba, Cordoba, Spain and CIBER Fisiopatologia Obesidad y Nutricion (CIBEROBN), Instituto de Salud Carlos III, Madrid, Spain

## References

[1] Talavera-Garcia E, Delgado-Lista J, Garcia-Rios A, Delgado-Casado N, Gomez-Luna P, Gomez-Garduño A, et al (2016) Influence of Obesity and Metabolic Disease on Carotid Atherosclerosis in Patients with Coronary Artery Disease (CordioPrev Study). PLoS ONE 11(4): e0153096 doi:10.1371/journal.pone.0153096 27064675 10.1371/journal.pone.0153096 PMC4827867

