# Supplementary material for: Influence of Obesity and Metabolic Disease on Carotid Atherosclerosis in Patients with Coronary Artery Disease (CordioPrev Study)
Source: PLoS One. 2016 Apr 11;11(4):e0153096. doi: 10.1371/journal.pone.0153096 (PMC4827867; doi:10.1371/journal.pone.0153096)
Supplement: S1 Fig — (DOCX) [file pone.0153096.s001.docx]

**Supplemental material:**


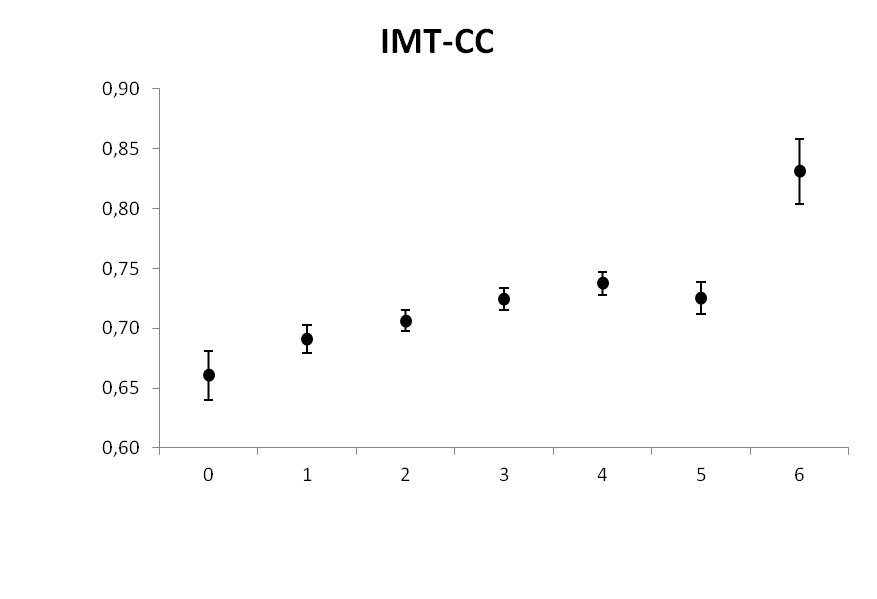


*

IMT-CC of patients in the CordioPrev study depending on the number of metabolic abnormalities. *p<0.05 versus any other group. Number of abnormalities correlated to IMT-CC (p = 2*10^-7^. r 0.42).
